# Guidelines at a crossroad: comparing European and American guidelines regarding the use of imaging in peripheral vascular arterial disease and aortic disease

**DOI:** 10.1093/ehjimp/qyae123

**Published:** 2024-11-27

**Authors:** Riccardo Liga, Aurelien Hostalrich, Alessia Gimelli, Jean-Baptiste Ricco

**Affiliations:** Dipartimento di Patologia Chirurgica, Medica, Molecolare e dell’Area Critica, Azienda Ospedaliera Universitaria Pisana, Via Paradiso, 56100 Pisa, Italy; Department of Vascular Surgery, Toulouse University Hospital, 2 Rue Charles Viguerie, 31300 Toulose, France; Department of Imaging, Fondazione Toscana Gabriele Monasterio, Via Moruzzi 1, 56100 Pisa, Italy; Académie Nationale de Médecine, 16 Rue Bonaparte, 75006 Paris, France

**Keywords:** cardiac imaging, guidelines, aortic diseases, peripheral vascular disease

## Abstract

This review examines the differences and similarities between the European and American guidelines concerning the use of imaging in the diagnosis and management of peripheral arterial disease (PAD) and aortic disease. PAD and aortic conditions contribute significantly to global cardiovascular morbidity and mortality; yet, they are often underdiagnosed and undertreated. Imaging plays a critical role in addressing this gap, with the European Society of Cardiology and American Cardiac Society offering different approaches to diagnostic and interventional imaging modalities. The review highlights that while both guidelines endorse duplex ultrasound as the first-line imaging method for PAD, discrepancies arise in the use of advanced modalities such as computed tomography angiography and magnetic resonance angiography. The European guidelines adopts a more conservative approach, reserving these advanced techniques for specific clinical scenarios, whereas the American guidelines places a stronger emphasis on comprehensive imaging for all patients with suspected PAD. The review also compares the guidelines on aortic disease, noting consensus on the role of computed tomography angiography and magnetic resonance angiography for aortic aneurysm diagnosis, but with differences in the emphasis on transoesophageal echocardiography, which is more strongly recommended by the American guidelines for acute cases. The manuscript calls for harmonization of these guidelines to streamline clinical practice and improve patient outcomes.

## Introduction

### Background and rationale

Peripheral vascular arterial disease (PAD) and aortic diseases are highly prevalent in the western world and contribute to significantly increased cardiovascular morbidity and mortality in the general population worldwide.^1,2^ The European Society of Cardiology (ESC) and the American Cardiac Societies have developed comprehensive guidelines^3–5^ to manage these conditions, both stating that intensive preventive strategies for both PAD and aortic disease are eagerly needed. However, as confirmed by available appraisals, a consistent underdiagnosis of these disease conditions is generally encountered,^6^ likely leading to patients’ undertreatment. While different cardiac and vascular imaging techniques are extensively used to unmask and assess the severity of PAD and aortic disease, a multi-modality imaging approach is frequently required to characterize such complex conditions that may share both atherosclerotic and non-atherosclerotic aetiologies. Despite the shared goal of improving patient outcomes, notable differences exist in the recommendations made by these organizations for the diagnosis and management of patients with both PAD and aortic disease, especially regarding imaging modalities used for these purposes.

### Scope of the review

This review aims to compare the European and American guidelines for the management of PAD and aortic diseases,^3,4^ focusing on the role of imaging modalities for disease diagnosis and patient management. By highlighting the similarities and discrepancies between the two authoritative statements, this review seeks to provide a comprehensive understanding of the guidelines, their clinical implications, and potential areas for harmonization.

### Objectives

The primary objectives of this review are

- to provide an overview of PAD and aortic diseases;
- to compare the imaging modalities recommended in the ESC and American guidelines for diagnosing and managing PAD and aortic diseases;
- to analyse the similarities and discrepancies between the use of imaging in these guidelines;
- to discuss the clinical implications of these differences; and
- to explore potential avenues for harmonizing the use of imaging in the two guidelines.

## Peripheral vascular arterial disease

### Overview of PAD

PAD is highly prevalent worldwide, involving close to 5–6% of the global population.^7^ Although PAD is similarly (or even more) represented in women than in men, the former tend to experience a consistent delay in diagnosis and treatment, leading to worse outcomes than men.^8^ The presence of PAD confers a three-fold greater risk for mortality than the absence of any disease and a trend towards worse risk for major ischaemic cardiovascular events compared with individuals with coronary artery disease alone.^9,10^ Both conventional (smoking, diabetes, dyslipidaemia, and hypertension the best characterized) and non-traditional (history of pregnancy complications and autoimmune disease) risk factors lead to the development and progression of PAD. Moreover, in the last decade, genetic factors leading to an increased propensity for PAD have been reported, together with some environmental variables (i.e. pollution) that will need further characterization.^3,4^ As a general rule, PAD encompasses conditions characterized by the obstruction of peripheral arteries, mainly leading to reduced blood flow to the limbs. It is commonly caused by atherosclerosis and can be asymptomatic for a long time, then potentially leading to claudication during exercise, and finally (in its most advanced stage) putting the limb at risk in the context of critical ischaemia that can lead to severe complications, including limb loss and cardiovascular events.

Initial clinical assessment with the ankle–brachial index (ABI): the ABI is a simple, non-invasive, test assessing the physiology of lower extremities haemodynamic. Although possibly limited in patients with long-standing diabetes or chronic kidney disease—whereby the presence of incompressible calcified vessels may lead to false results, the ABI still remains the cornerstone for the initial diagnosis of patients with PAD at large. The ABI is measured in each leg using a blood pressure cuff and a continuous-wave Doppler device as the ratio of the higher systolic pressure in the ipsilateral dorsalis pedis and posterior tibial arteries divided by the higher of the left and right brachial artery systolic pressures. When needed, additional physiological testing can be performed, such as exercise ABI testing and assessment of segmental pressures. An ABI ≤0.90 confirms PAD diagnosis, while for values >1.40 (‘non-compressible arteries’), assessing resting toe–brachial index (TBI) is essential for PAD diagnosis. In these patients, the TBI can be essential to diagnose the presence of chronic limb-threatening ischaemia (CLTI) that can be present. It must be considered that there are categories of patients who may have normal ABIs at rest and suffer from claudication, in which case exercise ABIs on a treadmill at a standardized speed may be indicated to confirm the positive diagnosis of their symptomatic PAD.

### Imaging modalities in PAD diagnosis

**Figure 1:**
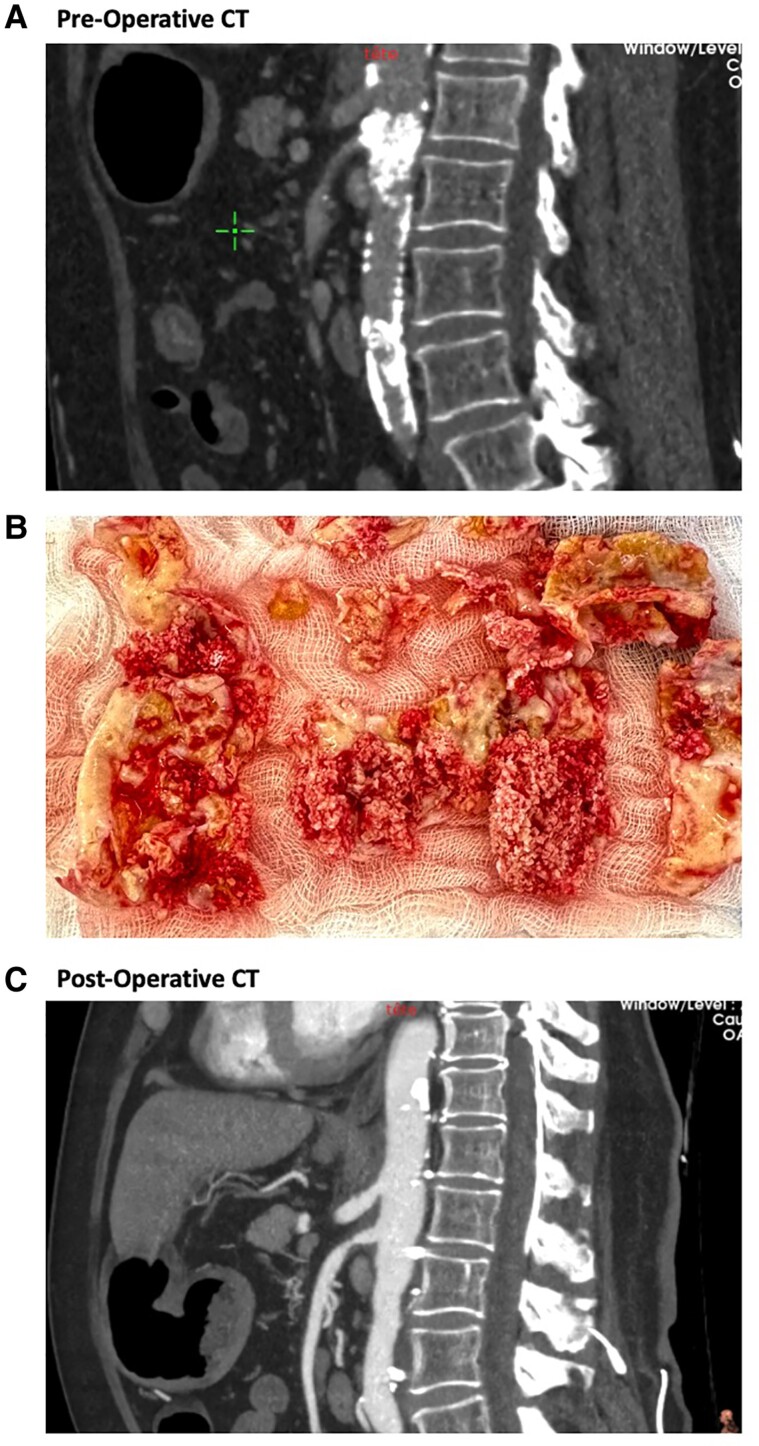
Representative computed tomography angiography images of a patient with coral reef on the abdominal aorta, before (*A*) and after (*C*) surgery as well as (*B*) intraoperative findings.

**Figure 2:**
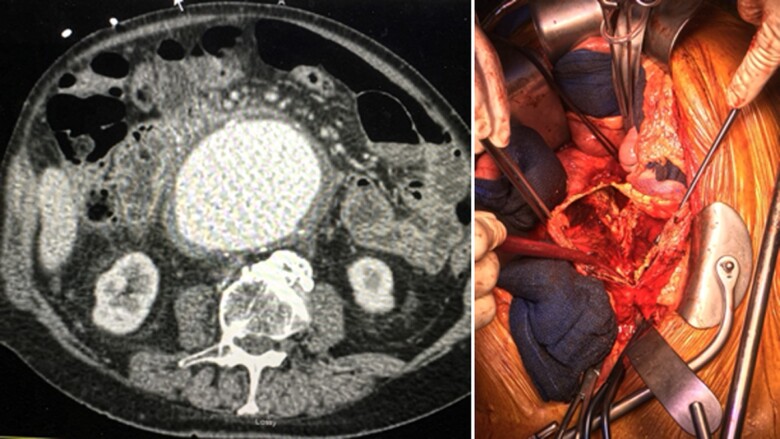
Computed tomography angiography transaxial image (left) and intraoperative (right) findings of a patients with a large abdominal aortic aneurysm.

**Figure 3:**
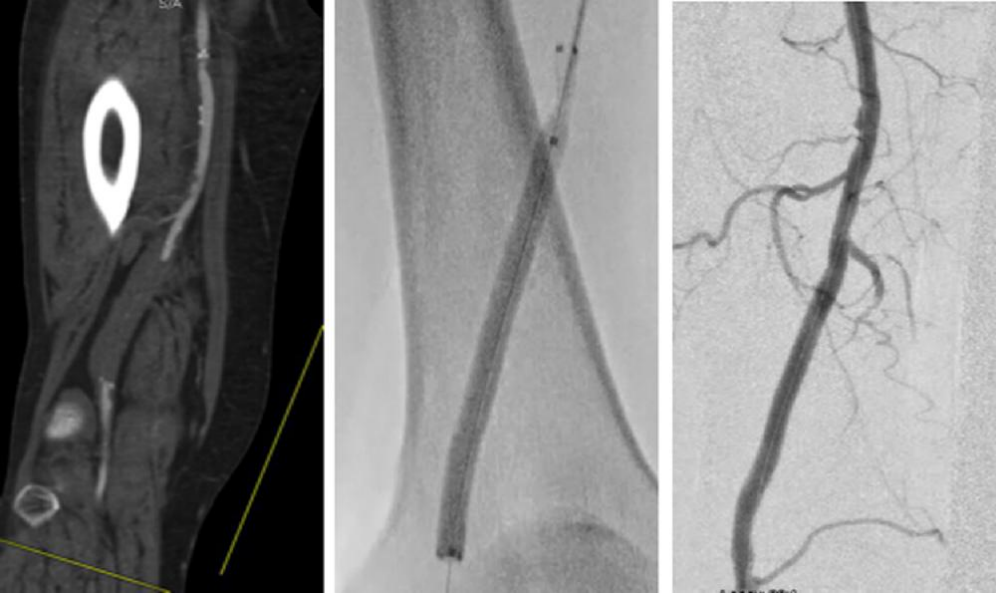
Computed tomography angiography (left panel) and digital subtraction angiography (middle and right panels) images obtained in a patient with intermittent claudication presenting an occlusion of the distal third of the superficial femoral artery, successfully treated with balloon angioplasty.

As detailed in both ESC and American guidelines, imaging plays a crucial role in diagnosing PAD, assessing disease severity, and planning interventions.^3,4^ The most used imaging modalities for the characterization of patients with PAD include the following:

- Duplex ultrasound (DUS) provides dynamic information on blood flow and vessel structure. It localizes both stenotic and aneurysmatic vascular lesions and objectively quantifies their extent and severity, providing a quantitative assessment of the haemodynamic relevance of a given stenosis that translates into excellent accuracy in unmasking significant vascular lesions (i.e. ∼90% sensitivity and specificity for detecting >50% luminal narrowing). Though some operator dependence should be acknowledged, DUS can easily detect subclinical atherosclerotic lesions, allowing earlier implementation of preventive strategies. More refined techniques, such as flow imaging and three-dimensional (3D) echography, could further improve DUS accuracy in selected clinical settings.
- Computed tomography angiography (CTA) offers detailed images of blood vessels and can identify stenosis and aneurysms. CTA has a better spatial resolution than magnetic resonance angiography (MRA) and better calcification visualization. CTA is the non-invasive reference standard for detecting and estimating vascular lesions. While severe vascular calcifications, particularly of distal arteries, have traditionally limited the accuracy of CTA in assessing stenosis severity, recent technological advancements (i.e. photon-counting CT devices) have considerably downgraded this limitation, allowing excellent image quality also in small, extensively calcified, vessels. Still, the possible long-term effect of ionizing radiation, coupled with the general possible contraindications to iodinated contrast medium (i.e. allergy and advanced kidney failure) should be considered before asking (possibly repeated) CTA studies (*Figure 1*).
- MRA, a non-invasive modality, provides high-resolution images of the arterial wall and lumen in conjunction with tissue and organ perfusion distal to the explored arterial lesion. MRA can provide data on plaque morphology and stenosis severity, with excellent accuracy (sensitivity and specificity >90%) in detecting significant lesions. Despite the still lower spatial resolution and the inability to visualize vascular calcifications, MRA is a reasonable alternative to CTA in the case of contraindications (*Figure 2*).
- Digital subtraction angiography (DSA) considered for many years as the gold standard for detailed vascular imaging, although invasive. While increasingly replaced by non-invasive imaging modalities for diagnostic purposes, DSA (possibly using carbon dioxide in patients with chronic kidney disease) remains to be used as part of an intention-to-treat strategy rather than for diagnostic purposes. Nevertheless, DSA remains a first-line examination in the context of below-the-knee (BTK) occlusions before any therapeutic action (*Figure 3*).

### Role of imaging in ESC guidelines

The ESC guidelines^3^ recommend the use of ABI and DUS as the first-line imaging modality for PAD diagnosis due to its non-invasive nature and accuracy in assessing blood flow and vessel structure. For more detailed imaging, CTA or MRA are recommended, especially for planning surgical or endovascular interventions.

#### Key recommendations in ESC guidelines

##### Ankle–brachial index

- ABI measurement is recommended for asymptomatic individuals aged ≥65 years and those with cardiovascular risk factors to detect early PAD and reclassify cardiovascular risk.
- Screening young low-risk patients (<65 years) without known cardiovascular risk factors using ABI is not routinely recommended but may be considered in the presence of significant risk factors.
- Exercise testing with post-exercise ABI measurements is recommended for patients with exertional limb pain and a resting ABI >0.90. A post-exercise ABI decrease of >20% can serve as PAD diagnostic criterion.
- Segmental limb pressures and pulse volume recording (PVR) are recommended for more detailed assessment of limb perfusion and to localize arterial occlusions or stenoses.

##### Duplex ultrasound

- DUS is recommended as the first-line imaging method to confirm PAD lesions (Grade IC).
- In symptomatic patients with aortoiliac or multi-segmental/complex disease, CTA and/or MRA are recommended as adjuvant imaging techniques for planning revascularization procedures (IC).
- As for ABI, DUS is recommended for patients’ follow-up after revascularization.
- Assessment of CLTI: In the presence of a clinical picture characterized by pain at rest and/or non-healing wounds as a result of severe PAD, comprehensive vascular imaging, including DUS, CTA, or MRA, is essential for evaluating revascularization options in patients with CLTI. In these patients, resting transcutaneous oxygen pressure (TcPO_2_) measurements are used to evaluate tissue viability.
- Non-invasive imaging, such as DUS, CTA, or MRA, is the cornerstone technique for the assessment of above-the-knee arteries. Due to the frequent presence of severe calcifications, BTK arteries may require DSA for final anatomical characterization and should be obtained to minimize the risk/extent of amputation.

In patients with CLTI, imaging of the entire affected limb should be considered (Grade IIAC). Patients with CLTI have ischaemia at rest, non-healing ulcers, or foot gangrene caused by severe hypoperfusion (ankle pressure <50 mmHg, toe pressure <30 mmHg, or TcPO_2_ < 30 mmHg).

#### Acute limb ischaemia

- CTA and DUS—with MRA frequently unfeasible in the emergency setting due to limited availability and relatively prolonged acquisition times—are all valuable options, based on local availability. DUS helps define treatment urgency, since the absence of both arterial and venous Doppler signals may indicate that the limb may be irreversibly damaged, but any decision to amputate should also consider clinical data and the duration of acute ischaemia. DSA is mostly not indicated for diagnostic purposes, while still a cornerstone for (interventional) therapeutic purposes.

### Role of imaging in American guidelines

The American guidelines^4^ similarly endorse ABI as the initial test for the evaluation of patients with suspected PAD. DUS can be used for PAD characterization. There is an emphasis on the use of CTA and MRA for comprehensive evaluation, particularly in patients who are candidates for any revascularization procedure. However, in patients with a confirmed diagnosis of PAD in whom revascularization is not being considered, CTA, MRA, or catheter angiography should not be performed solely for anatomic assessment.

#### Key recommendations in American guidelines

##### Resting ABI measurement

In patients with history or physical examination findings suggestive of PAD (Level 1 recommendation) or at risk for PAD (Level 2 recommendation), the resting ABI, with or without ankle PVRs and/or Doppler waveforms, is recommended to establish the diagnosis.

- In patients with PAD and an abnormal resting ABI (<0.90), the exercise treadmill ABI test can be useful to objectively assess the functional status and walking performance (Level 2a).
- Segmental leg pressures with PVR and/or Doppler waveforms are reasonable in patients with chronic symptomatic PAD (Level 2a). In patients with suspected CLTI, it is reasonable to perform additional perfusion assessments, including toe pressure/TBI, TcPO_2_, and skin perfusion pressure, which have shown the ability to assess local perfusion and determine wound-healing potential and risk for amputation (Level 2a).

##### Imaging tests

- In patients with suspected PAD and inconclusive ABI (±physiological testing)—such as non-healing wounds or exertional symptoms in patients unable to support a treadmill testing—imaging with DUS, CTA, or MRA may be considered to establish the diagnosis of PAD.

##### Chronic limb-threatening ischaemia

- The ABI alone may be inadequate to assess patients with suspected CLTI, in whom DUS, CTA, MRA, or catheter angiography, are useful to determine revascularization strategy (Level 1).
- The imaging approach for planning revascularization in patients with CLTI generally includes non-invasive imaging techniques (i.e. DUS, CTA, or MRA based on local availability and experience). However, for some patients with CLTI, proceeding directly to DSA may minimize the delay for revascularization.

### Discrepancies and similarities in imaging recommendations for PAD

Both the ESC and American guidelines^3,4^ agree on the primary use of DUS for initial diagnosis. The discrepancies lie in the subsequent choice of advanced imaging modalities, with the American guidelines placing more emphasis on the use of CTA and MRA compared with the ESC guidelines, which prefer a more conservative approach, reserving advanced imaging for specific clinical scenarios. Though possibly not exclusively, this discrepancy might be related to different training and practices between the US and EU vascular physicians, with the former having DUS more frequently performed by technologists rather than vascular physicians.

#### Diagnostic imaging

- Similarities: Both documents agree that diagnostic imaging plays a critical role in the initial assessment and diagnosis of PAD. The ABI is presented as a cornerstone diagnostic tool in both guidelines. When ABI is inconclusive, additional non-invasive imaging techniques, such as DUS, CTA, and MRA, are recommended to further define the anatomy and severity of PAD.
- Differences: The American guidelines emphasize the use of PVR and skin perfusion pressure, and toe pressure/TBI in conjunction with ABI for a more comprehensive physiological assessment.

#### Pre-operative imaging

- Similarities: Both sets of guidelines recommend the use of non-invasive imaging to plan revascularization procedures. DUS, CTA, and MRA are commonly used to map vascular anatomy and identify the precise location and severity of arterial lesions before surgery. Both guidelines recognize the importance of imaging during the peri-operative period, especially in guiding and confirming the success of revascularization procedures. Intraoperative DUS and angiography are standard practices to ensure that the interventions effectively address arterial blockages.
- Differences: The American guidelines^4^ discuss the individualized approach to selecting imaging modalities based on patient-specific factors and local resource availability. They also mention the potential of going directly to invasive catheter angiography in certain cases to avoid delays and minimize risks associated with additional imaging. The American guidelines^4^ specifically address the risks associated with peri-operative imaging modalities, such as exposure to ionizing radiation and contrast-induced nephropathy from CTA and catheter angiography. This discussion is less prominent in the ESC guidelines, which focus more on the technical aspects of the imaging techniques rather than the associated risks.

#### Post-operative imaging and follow-up

- Similarities: Both documents^3,4^ stress the importance of routine post-operative imaging to monitor the success of revascularization procedures and detect any complications early. DUS is frequently recommended for ongoing surveillance due to its non-invasive nature and effectiveness in evaluating graft patency and arterial health.
- Differences: The American guidelines^4^ provides a more detailed framework for post-operative follow-up, including specific intervals and the use of various imaging modalities depending on the clinical scenario. The ESC guidelines,^3^ while also recommending routine follow-up, does not elaborate as extensively on the specific intervals or the choice of imaging postoperative modalities.

## Aortic disease

### Overview of aortic disease

Aortic diseases encompass a range of conditions that can lead to significant morbidity and mortality due to the obvious critical role of this major vessel in systemic circulation. According to the ESC and American’s guidelines,^3,5^ we will consider aortic aneurysms and aortic dissections, each presenting unique challenges in diagnosis and management.

#### Aortic aneurysms

Aortic aneurysms are characterized by an abnormal dilation of the aorta, which can occur in the thoracic or abdominal segments. While the correct definition of an ‘aneurysm’ is still a matter of some debate, the evidence agrees in defining a thoracic aorta with a diameter of 4.0−4.4 cm as ‘dilated’, and ≥4.5 cm in size as an ‘aneurysm’.^3^ This classification is justified by the significant increase in the risk of aortic-related adverse events that are observed at follow-up as aortic diameter increases. Specifically, the risk of aortic rupture increases when the diameter is >5 cm, reaching 3.7% per year with aortic diameters >6 cm.^11^ However, a still sizable proportion of acute aortic syndromes occur in the presence of smaller vessels, with the rate of AD abruptly increasing for diameters ≥4.5 cm (i.e. >6000 times than ≤3.4 cm in size).^12^

Aortic aneurysms have both genetic and non-genetic causes, occurring sporadically or within families. Key risk factors include hypertension, atherosclerosis, inflammation, and smoking. Genetic conditions like Marfan syndrome, Ehlers–Danlos syndrome, and bicuspid aortic valve are linked to aneurysms, particularly in the aortic root and ascending aorta.^3,5^ Descending aortic aneurysms are often degenerative and occur later in life. Thoracic aortic aneurysms (TAAs) are usually asymptomatic until large or ruptured, requiring surveillance with imaging, such as echocardiography, CT, or MRI. Treatment ranges from monitoring to surgery, depending on size and symptoms.

#### Acute aortic syndromes

Acute aortic syndromes (AASs) are conditions in which there is an interruption in the integrity of the aortic vessel wall. AASs are commonly subdivided into three life-threatening conditions, namely aortic dissection, intramural haematoma, and penetrating atherosclerotic ulcer. An aortic dissection occurs when there is a tear in the intimal layer of the aorta, allowing blood to enter the aortic wall and create a false lumen. This condition is life-threatening and requires immediate medical attention. Aortic dissections are classified based on their location (Stanford Type A and Type B), with Type A involving the ascending aorta and Type B involving the descending aorta. The management of aortic dissection often involves blood pressure control, pain management, and surgical or endovascular intervention, particularly for Type A dissections, which necessitate emergency surgery.

### Imaging modalities in aortic disease diagnosis

Imaging is vital in diagnosing aortic diseases, also evaluating disease progression and planning interventions. Key imaging modalities include:

- Echocardiography: It is essential for the initial assessment and follow-up of aortic root and ascending aortic diseases. Transthoracic echocardiography (TTE) generally allows an adequate assessment of aortic root while the descending thoracic aorta is insufficiently visualized in a significant proportion of patients. As such, TTE is useful for initial evaluations of aortic disease and for follow-up of patients in the case of isolated aortic root disease (although a periodic assessment of the remaining vascular tree would be still needed).
- CTA: Thanks to multi-planar reconstructions, CTA provides detailed images of the entire aorta, making it indispensable for diagnosing aneurysms, and dissections, and for planning both surgical and endovascular repairs (EVARs). CTA is the preferred modality for detailed anatomical assessment due to its high resolution and ability to visualize the extent of disease and involvement of branch vessels.
- MRA: A non-invasive modality that offers high-resolution images without ionizing radiation, MRA is particularly useful for patients with contraindications to CT, such as those with renal insufficiency or allergies to iodinated contrast. It is also beneficial for serial follow-ups, particularly in younger patients (i.e. those with syndromic aortic aneurysms) and after open or endovascular interventions due to its lack of radiation exposure.
- Transoesophageal echocardiography (TEE): While more invasive, TEE provides excellent visualization of the aortic root and ascending aorta. It is particularly valuable in acute settings to quickly assess the proximal aorta and detect complications, such as dissections or involvement of the aortic valve. TEE provides superior visualization of the aortic root, and ascending and descending thoracic aorta, particularly when TTE is inconclusive. On the other hand, the aortic arch may be incompletely visualized by TEE, possibly leading to some degree of diagnostic uncertainty in the acute setting.

The selection of imaging modality often depends on the specific clinical scenario, patient condition, and the detailed anatomical information required for diagnosis and management. Each modality has unique advantages that contribute to comprehensive care in patients with aortic diseases.

### Role of imaging in ESC guidelines

The ESC guidelines^3^ advocates for the use of CTA and MRA as primary imaging modalities for diagnosing and monitoring aortic diseases. Echocardiography is recommended for initial evaluation and follow-up, especially in patients with aortic root and ascending aorta involvement.

#### Key recommendations in ESC guidelines

##### CTA and MRA for detailed assessment

CTA is the preferred imaging modality for detailed anatomical evaluation and surgical planning due to its high spatial resolution and ability to visualize vascular calcifications. It is particularly useful for diagnosing aneurysms, and dissections, and assessing pre- and post-operative aortic anatomy. MRA is recommended as an alternative for patients who have contraindications to CTA, such as those with renal insufficiency or allergies to iodinated contrast material. MRA provides high-resolution images without ionizing radiation and is effective in evaluating aortic wall pathology (i.e. in the case of aortitis).

##### Echocardiography for initial evaluation

TTE is useful for the initial evaluation of the aortic root and ascending aorta. It is a non-invasive and widely available imaging modality that provides essential information about aortic dimensions and the presence of aortic valve disease. However, TEE is recommended when TTE results are inconclusive. TEE offers superior visualization of the thoracic aorta, including detailed images of the aortic root, and thoracic aorta (less accurate concerning the aortic arch), making it a critical tool in both diagnostic and intraoperative settings.

##### Routine screening for aneurysms

Ultrasound screening for abdominal aortic aneurysms (AAAs) is recommended for men aged ≥65 years, and for women aged ≥65 with a history of smoking. This screening approach is based on substantial evidence indicating that early detection of AAA can significantly reduce the risk of rupture and subsequent mortality. The ESC guidelines emphasizes that screening should be considered particularly in individuals with a family history of AAA, given the increased risk associated with a genetic predisposition.

##### Follow-up imaging

Regular follow-up imaging with CTA or MRA is advised to monitor aneurysm size and growth rates. The ESC guidelines specifies that after the initial repair of TAAs, follow-up imaging should be performed within the first month, and then annually for the first 2 years. If the findings are stable, imaging intervals may be extended. For patients who have undergone EVAR of AAAs, follow-up imaging is recommended at 1 and 12 months post-operatively, followed by annual imaging if no abnormalities are detected. On each occasion, the diameter of the AAA should be precisely noted to monitor disease progression. These guidelines ensure early detection of complications such as endoleaks. In patients with aortic genetic diseases, periodic follow-up of the affected (dilated) aortic (or vascular) segments is prescribed (semi-annually or annually depending on the aortic diameter). Nevertheless, considering the frequently widespread effect of such diseases, a periodic assessment of the entire vascular tree is mandated (i.e. every 3–5 years).

### Role of imaging in American guidelines

The American^5^ guidelines similarly emphasizes the use of CTA and MRA for the comprehensive evaluation of aortic diseases. However, there is a greater emphasis on using TEE for detailed assessment of the aortic root and ascending aorta.

#### Key recommendations in American guidelines

- CTA and MRA for detailed assessment: CTA remains the preferred modality for detailed anatomic evaluation of the aorta, particularly useful for surgical planning due to its high resolution and ability to provide comprehensive views of the aortic structure. MRA is recommended as an alternative imaging technique for patients who have contraindications to CTA, such as those with renal insufficiency or contrast allergies.
- TEE for detailed evaluation: TEE is emphasized for its high-resolution imaging capabilities, especially useful for the detailed assessment of the aortic root and ascending aorta. This modality is particularly recommended in cases of acute aortic syndromes, where rapid and precise imaging can be critical for effective management.
- Follow-up imaging: Regular follow-up imaging is advised to monitor the size and growth rate of aortic aneurysms and dissections. In general terms, a semi-annual ultrasound assessment (if feasible) is advised after diagnosis to monitor the rate of growth of the dilated vascular segment. In the case of stable measures, annual surveillance is conventionally advised. The guidelines recommends using CTA or MRA for follow-up evaluations to ensure any changes in the condition are detected promptly. This ongoing monitoring is crucial for timely intervention and to prevent adverse outcomes associated with the progression of aortic disease.
- Routine screening for aneurysms: Similar to ESC guidelines,^3^ the American^5^ guidelines recommends routine ultrasound screening for AAA in men aged 65 years and older, and in women aged ≥65 with a history of smoking. This is to facilitate early detection and management of aneurysms to prevent rupture and associated complications.

#### Multi-disciplinary approach

The American guidelines^5^ also highlights the importance of a multi-disciplinary approach in managing patients with aortic disease. This includes the involvement of specialized aortic centres and teams that can provide comprehensive care, from initial diagnosis to surgical intervention and post-operative management. Such an approach is shown to improve patient outcomes, particularly in complex cases like acute Type A aortic dissection.

### Discrepancies and similarities in imaging recommendations for aortic disease

Both the ESC and American^3,5^ guidelines agree on the primary use of CTA and MRA for comprehensive evaluation of aortic diseases. The discrepancies are mainly in the use of TEE, with the American guidelines placing a stronger emphasis on its role in detailed assessment, especially in acute settings.

## Discrepancies and similarities in imaging recommendations for aortic disease

### Diagnostic imaging

#### Similarities

Both the ESC and American^3,5^ guidelines emphasize the importance of high-resolution imaging modalities for the accurate diagnosis of aortic diseases. Commonly recommended modalities include:

- Echocardiography (TTE and TEE): Both sets of guidelines agree on the use of TTE for the initial evaluation of the aortic root and ascending aorta. They also highlight the utility of TEE for more detailed imaging, particularly in cases where TTE results are inconclusive.
- CTA: It is universally recommended for its detailed anatomical visualization, which is crucial for diagnosing aneurysms, dissections, and other structural abnormalities. Its ability to provide comprehensive 3D reconstructions makes it a gold standard in both sets of guidelines.
- MRA: It is recognized as an essential tool for patients who cannot undergo CTA, such as those with renal insufficiency or allergies to iodinated contrast. Both guidelines appreciate MRA’s high-resolution images and its non-invasive nature without ionizing radiation.

#### Differences

While the overall recommendations are similar, there are nuanced differences in emphasis and additional considerations:

- ESC guidelines^3^: The guidelines places a stronger emphasis on genetic testing and counselling in conjunction with imaging, particularly for congenital aortic conditions. This reflects a broader approach that includes genetic predispositions and family history as integral to diagnosis and management.
- American guidelines^5^: The guidelines highlights the rapid assessment capabilities of TEE in acute aortic syndromes, suggesting its use more strongly in emergency settings. The American guidelines also underscores the role of specialized aortic centres in managing complex cases, advocating for a more centralized and multi-disciplinary approach.

### Pre-operative imaging

#### Similarities

Both guidelines stress the importance of comprehensive imaging to plan surgical or endovascular interventions effectively:

- Detailed anatomical assessment: CTA is the preferred modality for pre-operative planning due to its high resolution and ability to visualize vascular calcifications, branch vessels, and the extent of the disease.
- Use of MRA: For patients who cannot undergo CTA, MRA serves as a reliable alternative, providing detailed anatomical information necessary for surgical planning.

#### Differences

Differences arise in specific recommendations for certain patient populations and procedural contexts:

- ESC guidelines^3^ emphasizes the integration of imaging findings with genetic data and other risk factors in pre-operative planning, particularly for patients with known genetic syndromes affecting the aorta.
- American guidelines^5^ advocates for the use of TEE in the intraoperative setting for real-time guidance during surgery, especially in complex aortic repairs. This recommendation is particularly highlighted for acute Type A aortic dissection.

### Post-operative imaging and follow-up

#### Similarities

The follow-up and monitoring protocols show considerable alignment:

- Regular follow-up imaging: Both guidelines recommend regular imaging follow-up using CTA or MRA to monitor for aneurysm size, growth rates, and potential complications, such as endoleaks post-EVAR.
- Initial post-operative imaging: Initial follow-up imaging is advised within the first month post-surgery, with subsequent imaging intervals adjusted based on patient stability and findings.

#### Differences

Variations exist in the frequency and specific imaging strategies:

- ESC guidelines^3^ suggests a more structured follow-up protocol with specific intervals: at 1 and 6 months and then annually if stable. There is also a focus on long-term surveillance, particularly in patients with genetic conditions or complex aortic repairs.
- American guidelines^5^ highlights the importance of a multi-disciplinary approach in follow-up care, involving aortic centres and teams. This approach ensures that any changes in patient conditions are managed promptly and effectively. The American also recommends more frequent imaging in the first 2 years post-repair to ensure early detection of complications.

These similarities and differences in imaging recommendations reflect the overarching goal of both guidelines: to provide precise, timely, and effective diagnosis and management of aortic diseases through the best use of available imaging technologies.

## Conclusion

### Summary of findings

This review highlights the key similarities and differences between the ESC and American guidelines for PAD and aortic diseases. While there is a consensus on the importance of imaging in diagnosis and management, discrepancies exist in the choice and application of specific imaging modalities.

### Clinical implications

Understanding these differences is crucial for clinicians practicing in diverse healthcare settings. The choice of imaging modality can significantly impact patient outcomes, and awareness of guideline variations can aid in making informed decisions.

### Future directions

Harmonizing the guidelines and developing a unified approach to imaging in PAD and aortic diseases could improve patient care and streamline clinical practice. Further research and collaboration between international bodies are essential to achieve this goal.

## Data Availability

No new data were generated or analysed in support of this research.

## Lead author biography

Dr Alessia Gimelli is a cardiologist and nuclear medicine specialist. She is leading the Nuclear Cardiology Unit and the related training program in Fondazione Toscana G. Monasterio, Pisa, Italy. Dr Gimelli has deep experience in clinical research on cardiovascular diseases and cardiovascular imaging and has contributed to several international high-profile projects. Moreover, she has authored numerous articles in peer-reviewed medical journals, and she was part of the editorial board of some international journals with senior roles. In 2021, she won the WomenAsOne—2022 ESCALATOR AWARDS in the Mentor Match Category. Dr Gimelli has served in many different roles within the ESC over the past 20 years. She is the Editor in Chief of the *European Heart Journal—Imaging Methods and Practice*.
